# Prognostic nutritional indices and long-term survival after endoscopic submucosal dissection for early gastric cancer in elderly patients: a systematic review and meta-analysis

**DOI:** 10.3389/fnut.2026.1806717

**Published:** 2026-06-18

**Authors:** Huan Li, Xiao-Zhi Fu, Jie Chen, Xue-Fang Huang, Hui Long

**Affiliations:** Department of Gastroenterology, Tianyou Hospital Affiliated to Wuhan University of Science and Technology, Wuhan, China

**Keywords:** early gastric cancer, elderly patients, endoscopic submucosal dissection, long-term survival, nutritional indices

## Abstract

**Background:**

Nutritional risk and host vulnerability may influence long-term outcomes in elderly patients with early gastric cancer (EGC) undergoing endoscopic submucosal dissection (ESD). The prognostic nutritional index (PNI) and geriatric nutritional risk index (GNRI) are simple nutritional and prognostic indices, but their prognostic value in this population has not been systematically evaluated.

**Methods:**

We conducted a systematic review and meta-analysis of studies assessing the association between PNI or GNRI and long-term survival in elderly patients (≥ 65 years) treated with ESD for EGC. PubMed, Embase, and Web of Science were searched up to December 28, 2025. Hazard ratios (HRs) or comparable multivariable estimates with 95% confidence intervals (CIs) were synthesized using fixed- or random-effects models. Subgroup and sensitivity analyses were conducted to assess consistency and heterogeneity.

**Results:**

*Seven* studies involving 1,453 elderly patients were included. Low PNI was associated with poorer overall survival (pooled HR 2.91, 95% CI 2.12–3.99), with low heterogeneity. Lower GNRI was also associated with poorer survival outcomes (pooled estimate 2.87, 95% CI 1.36–6.02). Subgroup and sensitivity analyses supported the direction and robustness of the association for PNI.

**Conclusion:**

Lower PNI and GNRI were associated with poorer survival outcomes in elderly patients with EGC treated by ESD. These findings highlight the potential clinical relevance of nutritional risk assessment and may help inform risk stratification, but further prospective studies are needed to validate their clinical utility and assess whether targeted interventions can modify outcomes.

**Systematic review registration:**

identifier: CRD420251270408.

## Introduction

Gastric cancer remains a leading cause of cancer-related mortality worldwide, with particularly high incidence and mortality rates in East Asia (1). In recent years, broader access to screening and advances in endoscopic diagnostics have increased the proportion of patients diagnosed at an early stage (2). EGC generally refers to carcinoma confined to the mucosa or submucosa, irrespective of lymph node status (3). For selected patients with negligible or acceptable risk of lymph node metastasis, ESD is recommended as a minimally invasive treatment and can achieve en bloc and R0 resection while avoiding the physiological stress of gastrectomy (3, 4). These advantages are particularly relevant for elderly patients, in whom treatment tolerance, competing mortality, and the balance between procedural benefit and overall health status require individualized consideration (5, 6).

Post-ESD pathological curability is determined after resection and depends on margin status, lymphovascular invasion, invasion depth, histology, lesion size, and ulcerative findings (3). Curative ESD generally indicates that endoscopic resection is considered sufficient, whereas non-curative ESD indicates residual or lymph node metastasis risk and may require additional surgery or close surveillance. In elderly patients, however, ESD is sometimes performed under expanded, relative, or individualized indications because treatment decisions must balance oncologic risk against frailty, comorbidity burden, surgical tolerance, and life expectancy (6, 7).

Nutritional status is a clinically relevant component of systemic health and may reflect immune competence, inflammation, and tolerance to physiological stress. Malnutrition is common in older adults because of age-related declines in digestion, absorption, and metabolism, as well as disease-related anorexia and catabolism. It has been consistently associated with adverse outcomes in gastrointestinal cancers, including increased postoperative complications and reduced survival (8, 9). Simple anthropometric measures, such as body mass index (BMI), provide limited insight into immunonutritional status, prompting the use of composite indices. The Prognostic Nutritional Index (PNI), calculated from serum albumin levels and lymphocyte counts, was originally developed to estimate surgical risk in malnourished patients undergoing gastrointestinal surgery (10). The Geriatric Nutritional Risk Index (GNRI), derived from serum albumin and body weight relative to ideal body weight, was developed to evaluate nutrition-related risk in older adults (11). Accordingly, PNI and GNRI are clinically convenient indices reflecting nutritional risk and host vulnerability, and they have been widely used for prognostic assessment in older or oncologic populations (12, 13).

PNI and GNRI have been increasingly used as prognostic markers in surgical and oncologic settings. Previous meta-analyses have shown that low PNI is associated with poorer prognosis in patients with cancer, especially digestive system carcinomas, and in patients with gastric cancer undergoing surgery (14, 15). For GNRI, meta-analyses have also linked low GNRI with postoperative complications and worse long-term outcomes in gastrointestinal malignancies and gastric cancer (16, 17). However, their prognostic value after ESD in elderly patients with EGC remains less clearly defined. This population differs from conventional surgical cohorts because ESD is less invasive and is often selected for patients in whom survival may be influenced by age, comorbidity, functional reserve, competing mortality, and ESD curability status (5–7). Moreover, available studies differ in age thresholds, nutritional index cut-off values, follow-up duration, outcome definitions, and covariate adjustment. These differences limit the interpretation of individual findings and support the need for a quantitative synthesis.

To date, no systematic review or meta-analysis has quantitatively evaluated the prognostic value of PNI or GNRI for survival after ESD specifically in elderly patients with EGC. Given the increasing use of minimally invasive procedures in older populations and the unique vulnerabilities of this age group, a quantitative synthesis of existing evidence is warranted. In this study, we assessed the association between baseline PNI or GNRI and overall survival in elderly patients with EGC treated with ESD.

## Materials and methods

This meta-analysis was conducted in accordance with the Cochrane Handbook for Systematic Reviews of Interventions and is reported following the Preferred Reporting Items for Systematic Reviews and Meta-Analyses (PRISMA) guidelines (18, 19). The study protocol was prospectively registered in the PROSPERO database (registration number: CRD420251270408).

### Literature search

A systematic literature search was performed in PubMed, Embase, and Web of Science from database inception to December 28, 2025, to identify studies examining the association between nutritional indices and long-term outcomes in elderly patients with EGC treated with ESD. The search strategy incorporated both controlled vocabulary terms and free-text terms related to early gastric cancer, endoscopic submucosal dissection, and nutritional status, including the PNI and GNRI. No restrictions were applied regarding language or publication status. Reference lists of relevant articles were manually screened to identify additional eligible studies. The complete search strategy is detailed in Supplementary Table 1.

### Eligibility criteria

Studies were eligible for inclusion if they investigated elderly patients (aged ≥65 years) with histologically confirmed EGC treated with ESD and reported survival outcomes according to PNI or GNRI. Studies involving curative, non-curative, or mixed ESD populations were eligible. Overall survival was the primary outcome. Studies reporting short-term survival or death within a defined period after ESD were considered when they provided directly comparable lower-vs.-higher multivariable estimates for PNI or GNRI.

Studies were excluded if they included patients treated with primary surgical resection, palliative endoscopic procedures, or adjuvant chemotherapy or radiotherapy without a separate ESD cohort; focused on advanced gastric cancer, non-gastric malignancies, or experimental animal models; or assessed nutritional status using instruments other than PNI or GNRI. Case reports, case series, cross-sectional studies, reviews, editorials, and conference abstracts without sufficient data for quantitative synthesis were also excluded.

### Study selection and data extraction

Titles and abstracts were independently screened by two reviewers, followed by full-text assessment of potentially eligible studies. Disagreements were resolved through discussion, with consultation of a third reviewer when necessary. Data were independently extracted using a standardized data collection form, including study characteristics, patient demographics, ESD curability status, outcome definitions, nutritional indices, cut-off values, follow-up duration, effect measure, directly comparable lower-vs.-higher HRs or other comparable multivariable estimates with corresponding CIs, and covariates included in multivariable analyses. Adjusted estimates were preferentially extracted when available.

### Quality assessment

The quality of included studies was evaluated using the Newcastle–Ottawa Scale (NOS) (20, 21), which assesses participant selection, group comparability, and outcome ascertainment. Total scores range from 0 to 9, with studies scoring ≥7 considered high quality, 4–6 moderate quality, and < 4 low quality. Two reviewers independently performed the quality assessments, resolving discrepancies through discussion or consultation with a third reviewer. Particular attention was given to the definition of nutritional status, adjustment for confounders, validity of survival outcomes, and completeness of follow-up.

### Statistical analysis

HRs or comparable multivariable effect estimates with corresponding 95% CIs were synthesized to evaluate the association between nutritional indices and survival outcomes. Only directly comparable lower-vs.-higher multivariable estimates were included in the quantitative synthesis. Estimates were harmonized so that values greater than 1 indicated poorer survival in patients with lower PNI or GNRI. When different effect measures were included, the summary result was presented as a pooled estimate, and the effect measure and outcome definition were recorded.

Log-transformed effect estimates and their standard errors were used for meta-analysis (22). Reported estimates were extracted directly, and standard errors were calculated from the reported 95% CIs when they were not provided.

A fixed-effects model was used when statistical heterogeneity was low, whereas a random-effects model was applied when substantial heterogeneity was present (*I*^2^ > 50%). Heterogeneity was assessed using Cochran's Q test and quantified with the *I*^2^ statistic, with values of 0%−25%, 26%−50%, and >50% interpreted as low, moderate, and substantial heterogeneity, respectively (23). Prespecified subgroup analyses were conducted according to PNI cut-off value, mean age, proportion of male patients, and follow-up duration. Leave-one-out sensitivity analyses were performed when sufficient studies were available.

Publication bias was assessed using funnel plots and Egger's regression test when at least ten studies were available for a given outcome (24, 25). All statistical analyses were conducted using Review Manager (RevMan) software, version 5.4 (Cochrane Collaboration, Oxford, UK), and Stata software, version 14.0 (Stata Corporation, College Station, TX). All tests were two-tailed, with *P* < 0.05 considered statistically significant.

## Results

### Basic characteristics and quality assessment

**Figure 1:**
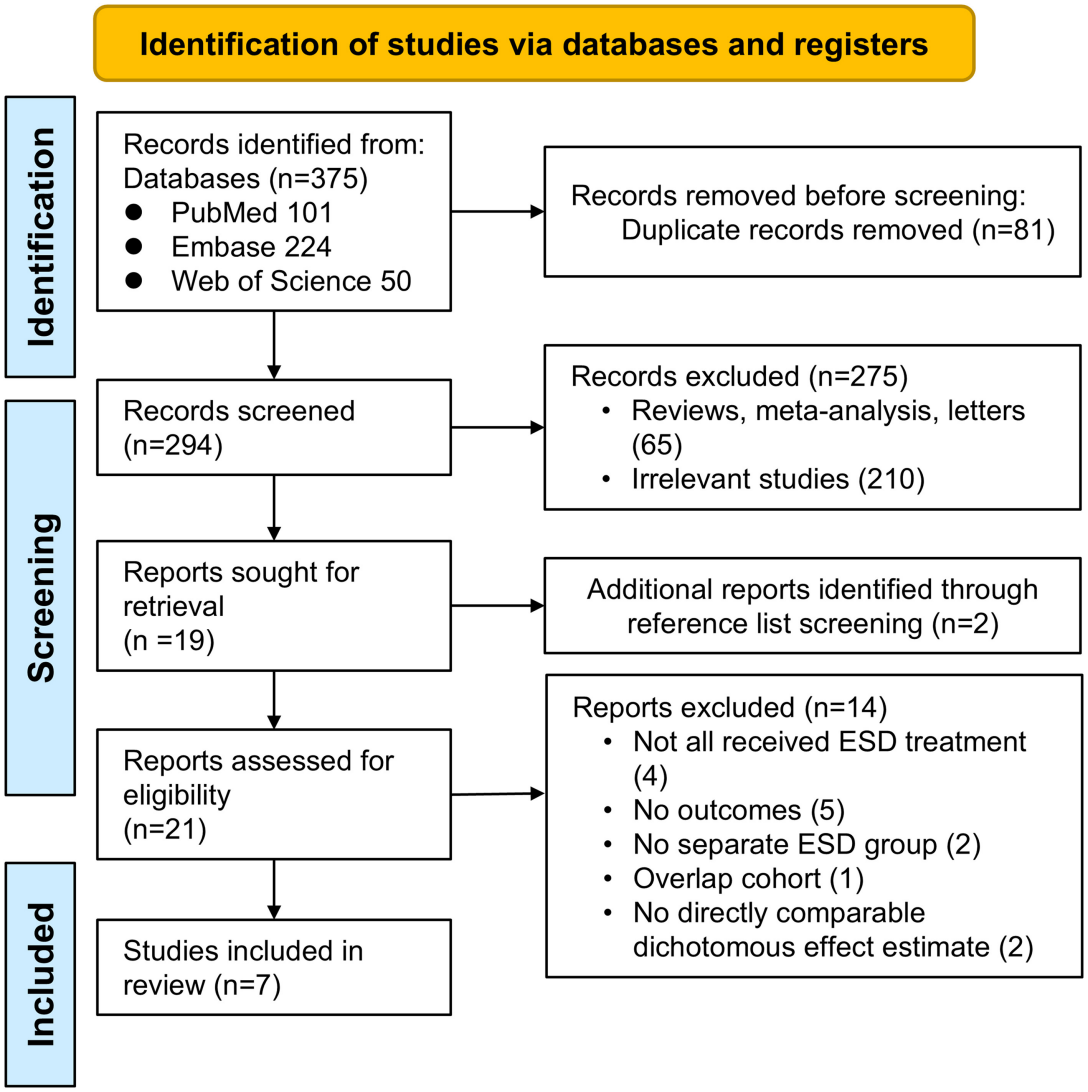
PRISMA flowchart presenting the study selection process for this systematic review and meta-analysis.

The systematic search identified 375 records from PubMed, Embase, and Web of Science. After removing 81 duplicates, 294 records remained for title and abstract screening. Of these, 275 were excluded for irrelevance or failure to meet inclusion criteria. Full texts of 21 potentially eligible articles were retrieved, including two identified through reference list screening. After detailed evaluation, 14 studies were excluded, including one because of cohort overlap and two because only continuous PNI or GNRI estimates without directly comparable lower-vs.-higher effect estimates were available. Ultimately, seven studies were included in the meta-analysis. The study selection process is illustrated in the PRISMA flow diagram (Figure 1).

A total of seven studies comprising 1,453 elderly patients with EGC treated by ESD were included (26–32). The mean of the reported mean or median ages was 78.4 years, and the median follow-up duration across studies was 58.0 months. Six studies were conducted in Japan, and one in China. Nutritional status was assessed using PNI in six studies and GNRI in two studies, with one study reporting both indices. PNI was calculated as 10 × serum albumin (g/dL) + 0.005 × lymphocyte count (/mm3), and GNRI as 14.89 × serum albumin (g/dL) + 41.7 × body mass index/22. Detailed study characteristics are presented in Table 1.

**Table 1:** Characteristics of the included studies.

Study	Country	ESD curabilitystatus^#^	Outcome definition	Study design	Total sample size	Mean age (years)	Male (%)	Median follow-up (months)	Total deaths (*n*)	Nutritionalassessment tool	Cut-off value	Effect measure	Effect estimate (95% CI)	Variables adjusted
Sekiguchi et al. (26)	Japan	Mixed	Overall survival	Retro	108	86.0	75.9	40.2	23	PNI	44.6	Adjusted HR	7.00 (2.20, 22.90)	Sex, ASA-PS, BMI, CCI, mGPS, PNI, NLR, etc.
Iwai et al. (27)	Japan	Mixed	Overall survival	Retro	109	83.0	64.2	58.0	21	PNI	47.7	Adjusted HR	3.44 (2.00, 5.90)	Age, ECOG PS, CCI, PNI, use of antithrombotic agents
Toya et al. (28)	Japan	Non-curative	Overall survival	Retro	87	78.0	74.7	80.4	27	PNI	44.8	Multivariable HR	1.50 (0.60, 3.77)	Age, ECOG PS, CCI, NLR
Toya et al. (30)	Japan	Mixed	Overall survival	Retro	70	86.0	60.0	72.0	33	PNI	42.5	Adjusted HR	3.40 (1.47, 7.86)	Not explicitly
Shi et al. (32)	China	Mixed	Overall survival	Retro	206	66.0	64.5	50.0	15	PNI	45.9	Adjusted HR	1.66 (0.54, 5.09)	ACCI, PNI, NLR, PLR, endoscopic curability
Hisada et al. (31)	Japan	Mixed	Overall survival	Retro	767	73.6	72.9	56.1	54	PNI GNRI	44.6/ 92	Adjusted HR	2.68 (1.43, 5.03) 3.08 (1.40, 6.76)	PS, CCI, PNI, GNRI, muscle status
Shimada et al. (29)	Japan	Non-curative	Death within 3 years after ESD/short-term survival	Retro	106	76.0	78.0	89.0	39	GNRI	92	Multivariable OR	1.60 (0.17, 15.00)^*^	Morphology, risk for LNM, comorbidity, ADL, habitation, NLR, GNRI, CRP

*Retro, retrospective study; PNI, prognostic nutritional index; GNRI, geriatric nutritional risk index; HRs, hazard ratios; Cis, confidence intervals; ASA-PS, American Society of anesthesiologists physical status; BMI, body mass index; CCI, Charlson comorbidity index; mGPS, modified Glasgow prognostic score; NLR, neutrophil-to-lymphocyte ratio; LNM, lymph node metastasis; ADL, activities of daily living; CRP, C-reactive protein; ECOG-PS, Eastern cooperative oncology group performance status; OR: odds ratio* ^#^ *In the ESD curability status column, “Mixed” indicates mixed curative/non-curative ESD populations, and “Non-curative” indicates non-curative ESD populations* ^*^ *Reported as a non-HR multivariable OR*

NOS scores ranged from 6 to 9 points. Two studies scored 6 points, four scored 7 points, and one scored 9 points (Table 2).

**Table 2:** Newcastle–Ottawa score for risk-of-bias assessment of the included studies.

Study	Representativeness of the exposed cohort	Selection of the non-exposed cohort	Ascertainment of exposure	Outcome not present at baseline	Control for age and sex	Control for confounding factors	Assessment of outcome	Enough long follow-up duration	Adequacy of follow-up of the cohort	Total
Sekiguchi et al. (26)	1	0	1	1	1	1	1	1	0	7
Iwai et al. (27)	1	0	1	1	1	1	1	1	0	7
Toya et al. (28)	0	0	1	1	1	1	1	1	0	6
Toya et al. (30)	0	0	1	1	1	1	1	1	0	6
Shi et al. (32)	1	0	1	1	1	1	1	1	0	7
Hisada et al. (31)	1	1	1	1	1	1	1	1	1	9
Shimada et al. (29)	1	0	1	1	1	1	1	1	0	7

### Prognostic value of PNI

**Figure 2:**
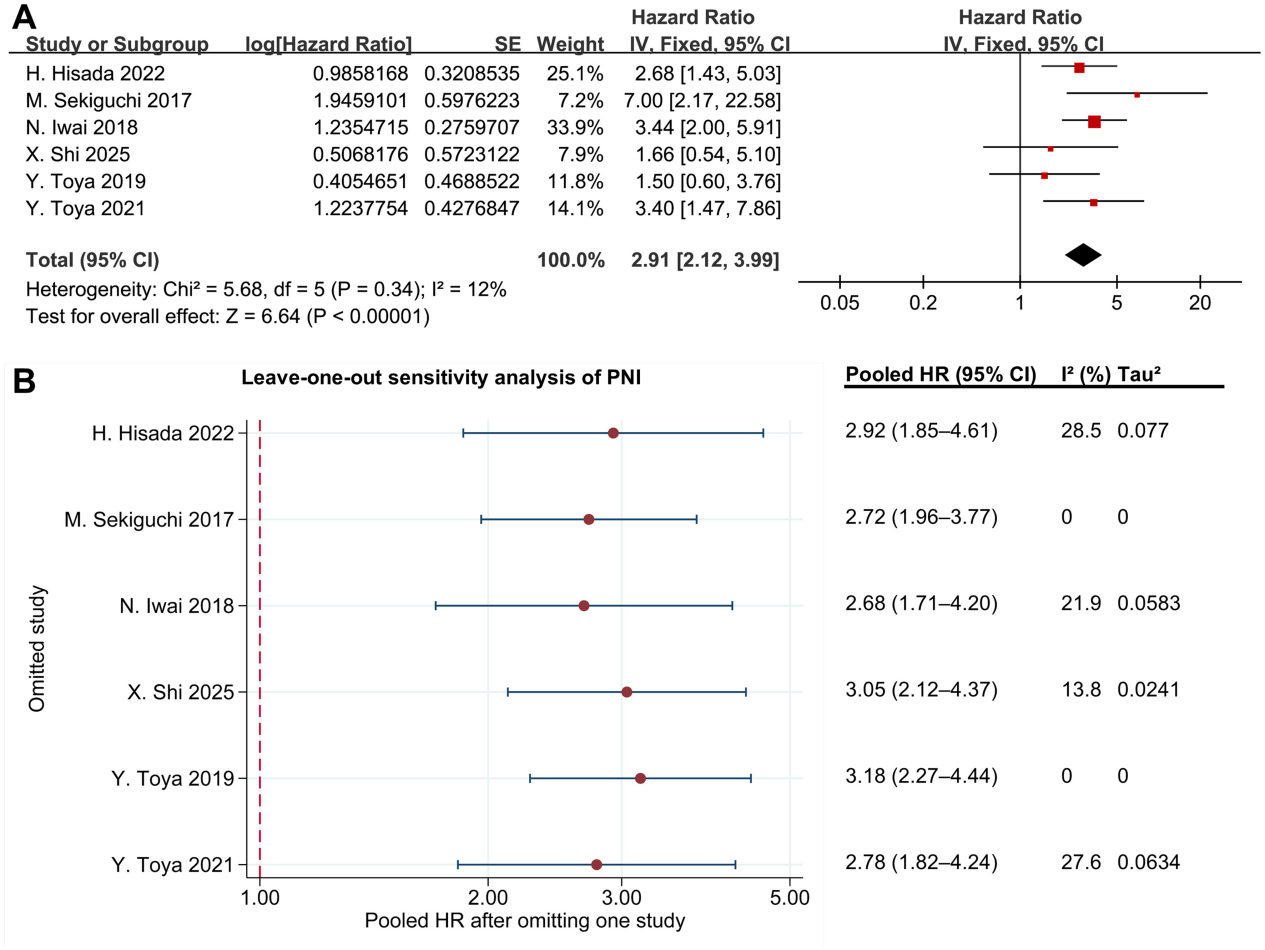
Forest plots for the association between prognostic nutritional index (PNI) and overall survival in elderly patients with early gastric cancer (EGC) treated with endoscopic submucosal dissection (ESD). **(A)** Primary analysis using a fixed-effects model; **(B)** leave-one-out sensitivity analysis.

Six studies evaluated the prognostic significance of PNI (26–28, 30–32). Reported PNI cut-off values ranged from 42.5 to 47.7, with a mean of 45.0. Using a fixed-effects model, low PNI was associated with poorer overall survival, with a pooled HR of 2.91 (95% CI, 2.12–3.99; *P* < 0.00001; Figure 2A), and low heterogeneity was observed (*I*^2^ = 12%).

Leave-one-out sensitivity analysis showed that the pooled HRs ranged from 2.68 (95% CI, 1.71–4.20) to 3.18 (95% CI, 2.27–4.44), and all estimates remained statistically significant. Heterogeneity remained low to moderate across analyses, with *I*^2^ values ranging from 0.0 to 28.5% (Figure 2B).

### Subgroup analyses

**Figure 3:**
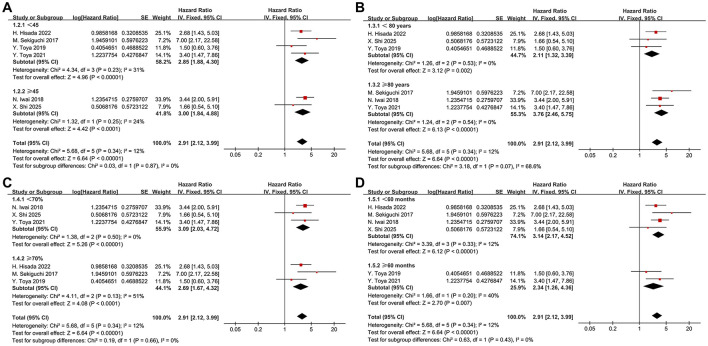
Forest plots for subgroup analyses of PNI and overall survival in elderly patients with EGC treated with ESD. **(A)** Subgroup analysis according to PNI cut-off value (< 45 vs. ≥45); **(B)** subgroup analysis according to mean age (< 80 vs. ≥80 years); **(C)** subgroup analysis according to the proportion of men (< 70% vs. ≥70%); **(D)** subgroup analysis according to follow-up duration (< 60 vs. ≥60 months).

Subgroup analyses were performed according to PNI cut-off value, mean age, proportion of male patients, and follow-up duration. In studies using a PNI cut-off value of less than 45 (26, 28, 30, 31), low PNI was associated with poorer overall survival, with a pooled HR of 2.85 (95% CI, 1.88–4.30; *P* < 0.00001; Figure 3A), and low-to-moderate heterogeneity was observed (*I*^2^ = 31%). In studies using a PNI cut-off of 45 or higher (27, 32), the pooled HR was 3.00 (95% CI, 1.84–4.88; *P* < 0.0001), with low heterogeneity (*I*^2^ = 24%). The test for subgroup differences was not statistically significant.

In studies with a mean age below 80 years (28, 31, 32), lower PNI remained associated with poorer survival, yielding a pooled HR of 2.11 (95% CI, 1.32–3.39; *P* = 0.002; Figure 3B), with no heterogeneity (*I*^2^ = 0%). In studies with a mean age of 80 years or older (26, 27, 30), low PNI was associated with a pooled HR of 3.76 (95% CI, 2.46–5.75; *P* < 0.00001), with no heterogeneity (*I*^2^ = 0%). The test for subgroup differences suggested a borderline trend toward variation between age categories (*P* = 0.07).

In studies with a male proportion below 70% (27, 30, 32), low PNI was associated with poorer survival, with a pooled HR of 3.09 (95% CI, 2.03–4.72; *P* < 0.00001; Figure 3C) and no heterogeneity (*I*^2^ = 0%). In studies with a male proportion of 70% or higher (26, 28, 31), the pooled HR was 2.69 (95% CI, 1.67–4.32; *P* < 0.0001), with substantial heterogeneity also observed (*I*^2^ = 51%). The test for subgroup differences was not statistically significant.

In studies with a median follow-up duration of less than 60 months (26, 27, 31, 32), low PNI was associated with poorer survival, with a pooled HR of 3.14 (95% CI, 2.17–4.52; *P* < 0.00001; Figure 3D), and low heterogeneity (*I*^2^ = 12%). In studies with a median follow-up of 60 months or longer (28, 30), the pooled HR was 2.34 (95% CI, 1.26–4.36; *P* = 0.007), with moderate heterogeneity (*I*^2^ = 40%). The test for subgroup differences was not statistically significant.

### Prognostic value of GNRI

**Figure 4:**
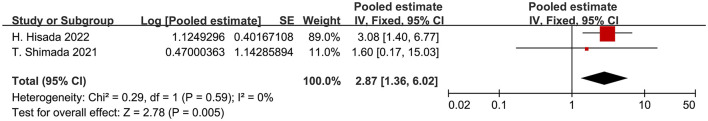
Forest plot for the association between GNRI and survival outcomes in elderly patients with EGC treated with ESD.

Two studies evaluated the association between GNRI and survival outcomes (29, 31), both using GNRI < 92 as the cut-off. Using a fixed-effects model, lower GNRI was associated with poorer survival outcomes (pooled estimate, 2.87; 95% CI, 1.36–6.02; *P* = 0.005; Figure 4), with no statistical heterogeneity (*I*^2^ = 0%).

### Publication bias

Publication bias was not formally assessed because fewer than 10 studies were available for each outcome, making funnel plot interpretation and Egger's regression unreliable.

### Discussion

In this meta-analysis of seven studies including 1,453 elderly patients with EGC treated by ESD, lower PNI or GNRI was associated with poorer survival outcomes. Low PNI was associated with a pooled HR of 2.91 (95% CI, 2.12–3.99), while lower GNRI was associated with a pooled estimate of 2.87 (95% CI, 1.36–6.02). The association between low PNI and poorer survival was stable in leave-one-out sensitivity analyses. These findings suggest that nutritional indices may help identify elderly patients at higher risk of reduced long-term survival after ESD.

Our findings are broadly consistent with previous gastric cancer studies. A meta-analysis of patients undergoing gastrectomy showed that low preoperative PNI was associated with poorer overall survival (33), and Zhang et al. (34) reported a similar prognostic role for PNI in elderly patients undergoing gastric cancer surgery (34). Evidence for GNRI has also been reported in gastric cancer populations. Miyamoto et al. (35) found that GNRI was associated with prognosis in patients aged ≥75 years with gastric cancer (35), while An et al. (36) identified GNRI as an important determinant of overall survival in stage I–III gastric cancer (36). However, most of these data come from surgical cohorts. Compared with gastrectomy, ESD is less invasive and is often selected for older patients whose prognosis may be shaped by age, comorbidity, functional reserve, and competing mortality. The prognostic meaning of PNI and GNRI after ESD should therefore be interpreted in this specific clinical context.

Overall survival after ESD in elderly patients may not mainly reflect gastric cancer control. Several included studies reported few gastric cancer-specific deaths despite long-term follow-up, suggesting an important role for non-cancer mortality (29, 30). In the multicenter study by Yoshikawa et al. (37), 3-year survival was associated with age-related and general health factors, including ECOG performance status, Charlson Comorbidity Index, and GNRI, rather than endoscopic curability alone (37). This pattern suggests that PNI and GNRI may reflect host condition rather than tumor biology alone. They may therefore be better viewed as geriatric-prognostic markers of physiological vulnerability.

This interpretation is supported by studies linking nutritional indices with frailty-related or inflammatory status. In an ESD cohort, Hisada et al. found that low PNI, low GNRI, and sarcopenia-related muscle parameters were associated with poorer overall survival, suggesting that nutritional indices may capture broader physical vulnerability beyond nutritional depletion alone (31). Miyamoto et al. reported that GNRI and the neutrophil-to-lymphocyte ratio were useful prognostic biomarkers in older patients with gastric cancer, indicating that nutritional and inflammatory status may jointly contribute to prognosis (35). Additional ESD evidence also supports this interpretation. Kim et al. reported that comorbidities, sarcopenia, and nutritional status were associated with long-term outcomes after ESD in patients aged ≥80 years (38). Thus, low PNI or GNRI may reflect a combined burden of nutritional risk, inflammation, impaired immune status, comorbidity, and reduced physiological reserve.

Clinical heterogeneity should also be considered. The included studies differed in age thresholds, follow-up duration, nutritional cut-off values, ESD curability status, outcome definitions, and adjustment variables. Some cohorts included mixed curative and non-curative ESD populations, while others focused on non-curative ESD. These differences may influence effect estimates, as non-curative ESD can affect subsequent treatment decisions, follow-up intensity, and competing risks. Even so, the direction of association was generally consistent. The limited heterogeneity observed in the primary PNI analysis and the stable leave-one-out sensitivity results suggest that the association was not driven by a single study. Residual variability across subgroup analyses may still reflect differences in cut-off values, age distribution, curability status, and patient selection.

From a clinical perspective, PNI and GNRI may have potential as simple adjunctive markers for pre-treatment risk stratification. Both indices are based on routinely available laboratory or anthropometric variables. In elderly patients being considered for ESD, low PNI or GNRI may be considered alongside established clinical and geriatric factors when assessing nutritional and functional vulnerability, rather than serving as stand-alone triggers for management changes. Evidence from broader elderly surgical populations also suggests that poor GNRI is associated with postoperative complications and poorer survival outcomes (39). Nutritional and immunonutritional strategies may improve selected perioperative outcomes in geriatric surgical settings, but their effect on long-term survival after ESD for EGC remains uncertain (40). The present findings should therefore be viewed as highlighting the potential clinical relevance of nutritional risk assessment rather than establishing that nutritional intervention improves survival.

Several limitations should be acknowledged. First, all included studies were observational, and residual confounding cannot be excluded. Adjustment variables differed across cohorts and were incompletely reported in some studies. Frailty, functional status, sarcopenia, comorbidity burden, endoscopic curability, and management after non-curative ESD were not consistently controlled. Selection bias, survivor bias, center-specific practice patterns, and outcome misclassification may also have influenced the estimates. Second, the GNRI analysis included only two studies, and one reported a non-HR multivariable estimate for short-term survival. Therefore, the pooled GNRI estimate should be interpreted cautiously. Third, publication bias could not be formally assessed because fewer than 10 studies were available for each outcome. Finally, most studies were conducted in East Asia, which may limit generalizability. In addition, because several studies were conducted in Japan and involved elderly ESD-treated populations, unrecognized cohort overlap cannot be completely excluded.

Future research should validate PNI and GNRI cut-off values in larger multicenter cohorts and across different regions. Studies using standardized outcome definitions, consistent adjustment for frailty and comorbidity, and detailed reporting of ESD curability status are needed. Combining nutritional indices with sarcopenia, frailty, inflammatory markers, or comprehensive geriatric assessment may warrant further evaluation for improving prognostic assessment. Prospective studies are also needed to determine whether nutritional support, prehabilitation, or geriatric interventions can modify outcomes in elderly patients with EGC undergoing ESD.

## Conclusion

Lower PNI and GNRI were associated with poorer survival outcomes in elderly patients with EGC treated by ESD. These indices may serve as simple prognostic markers reflecting nutritional risk and physiological vulnerability. Given the observational nature and clinical heterogeneity of the evidence, the findings should be interpreted as associations rather than proof of causality. Further prospective studies are needed to clarify whether nutritional or geriatric interventions can improve outcomes in this population.

## Data Availability

The original contributions presented in the study are included in the article/Supplementary material, further inquiries can be directed to the corresponding authors.
