# Supplementary material for: Prognostic nutritional indices and long-term survival after endoscopic submucosal dissection for early gastric cancer in elderly patients: a systematic review and meta-analysis
Source: Front Nutr. 2026 Jun 18;13:1806717. doi: 10.3389/fnut.2026.1806717 (PMC13322933; doi:10.3389/fnut.2026.1806717)
Supplement: Supplementary file 1 [file Table_1.docx]

**Supplementary Table 1** The search strategy of each database

| **Database** | **Search strategy** | **Results** |
| --- | --- | --- |
| **PubMed** | #1 "Stomach Neoplasms"[Mesh] OR "Early Gastric Cancer"[tiab] OR "Early Stomach Cancer"[tiab] OR “stomach tumor"[tiab] OR "Gastric Cancer, Early"[tiab] OR “neoplasm, stomach”[tiab] OR “stomach neoplasm”[tiab] OR “neoplasms, stomach”[tiab] OR “gastric neoplasms”[tiab] OR “gastric neoplasm”[tiab] OR “neoplasm, gastric”[tiab] OR “neoplasms, gastric”[tiab] OR “cancer of stomach”[tiab] OR “stomach cancers”[tiab] OR “gastric cancer”[tiab] OR “cancer, gastric”[tiab] OR “cancers, gastric”[tiab] OR “gastric cancers”[tiab] OR “stomach cancer”[tiab] OR “cancer, stomach”[tiab] OR “cancers, stomach”[tiab] OR “cancer of the stomach”[tiab] OR “gastric cancer”[tiab] | 154242 |
|  | #2 "endoscopic submucosal dissection" OR ESD OR "endoscopy" | 203409 |
|  | #3 "Nutritional Status"[Mesh] OR malnutrition OR undernutrition OR "nutritional risk" OR "prognostic nutritional index" OR "prognostic nutrition index" OR PNI OR "geriatric nutritional risk index" OR "geriatric nutrition risk index" OR GNRI OR "nutritional assessment" OR "nutritional screening" OR "nutritional indices" | 238523 |
|  | #4 #1 and #2 and #3 | **101** |
| **Embase** | #1 'stomach neoplasms'/exp OR 'stomach tumor'/exp OR 'early gastric cancer':ti,ab OR 'early stomach cancer':ti,ab OR 'gastric cancer, early':ti,ab OR 'neoplasm, stomach':ti,ab OR 'stomach neoplasm':ti,ab OR 'neoplasms, stomach':ti,ab OR 'gastric neoplasms':ti,ab OR 'gastric neoplasm':ti,ab OR 'neoplasm, gastric':ti,ab OR 'neoplasms, gastric':ti,ab OR 'cancer of stomach':ti,ab OR 'stomach cancers':ti,ab OR 'gastric cancer':ti,ab OR 'cancer, gastric':ti,ab OR 'cancers, gastric':ti,ab OR 'gastric cancers':ti,ab OR 'stomach cancer':ti,ab OR 'cancer, stomach':ti,ab OR 'cancers, stomach':ti,ab OR 'cancer of the stomach':ti,ab | 245802 |
|  | #2 'endoscopic submucosal dissection'/exp OR 'endoscopic submucosal dissection' OR esd OR endoscopy | 421580 |
|  | #3 'nutritional status'/exp OR malnutrition OR undernutrition OR 'nutritional risk' OR 'prognostic nutritional index' OR 'prognostic nutrition index' OR pni OR 'geriatric nutritional risk index' OR 'geriatric nutrition risk index' OR gnri OR 'nutritional assessment' OR 'nutritional screening' OR 'nutritional indices' | 247612 |
|  | #4 #1 AND #2 AND #3 | 354 |
|  | #4 #1 AND #2 AND #3 AND ('article'/it OR 'article in press'/it) AND [humans]/lim | **224** |
| **Web of Science** | TS =(("Stomach Neoplasms" OR "Early Gastric Cancer" OR "Early Stomach Cancer" OR "Gastric Cancer, Early" OR "stomach tumor" OR "neoplasm, stomach" OR "stomach neoplasm" OR "neoplasms, stomach" OR "gastric neoplasms" OR "gastric neoplasm" OR "neoplasm, gastric" OR "neoplasms, gastric" OR "cancer of stomach" OR "stomach cancers" OR "gastric cancer" OR "cancer, gastric" OR "cancers, gastric" OR "gastric cancers" OR "stomach cancer" OR "cancer, stomach" OR "cancers, stomach" OR "cancer of the stomach") AND ("endoscopic submucosal dissection" OR ESD OR "endoscopy") AND ("Nutritional Status" OR malnutrition OR undernutrition OR "nutritional risk" OR "prognostic nutritional index" OR "prognostic nutrition index" OR PNI OR "controlling nutritional status" OR "controlling nutrition status" OR CONUT OR "geriatric nutritional risk index" OR "geriatric nutrition risk index" OR GNRI OR "nutritional assessment" OR "nutritional screening" OR "nutritional indices")) | **50** |
